# BIX01294, an inhibitor of histone methyltransferase, induces autophagy-dependent differentiation of glioma stem-like cells

**DOI:** 10.1038/srep38723

**Published:** 2016-12-09

**Authors:** Iwona Anna Ciechomska, Piotr Przanowski, Judyta Jackl, Bartosz Wojtas, Bozena Kaminska

**Affiliations:** 1 Laboratory of Molecular Neurobiology, Neurobiology Center, Nencki Institute of Experimental Biology of Polish Academy of Science, 3 Pasteur Str., 02-093 Warsaw, Poland

## Abstract

Glioblastoma (GBM) contains rare glioma stem-like cells (GSCs) with capacities of self-renewal, multi-lineage differentiation, and resistance to conventional therapy. Drug-induced differentiation of GSCs is recognized as a promising approach of anti-glioma therapy. Accumulating evidence suggests that unique properties of stem cells depend on autophagy. Here we demonstrate that BIX01294, an inhibitor of a G9a histone methyltransferase (introducing H3K9me2 and H3K27me3 repressive marks) triggers autophagy in human glioma cells. Pharmacological or genetic inhibition of autophagy decreased LC3-II accumulation and GFP-LC3 punctation in BIX01294-treated cells. GSCs-enriched spheres originating from glioma cells and GBM patient-derived cultures express lower levels of autophagy related (*ATG*) genes than the parental glioma cell cultures. Typical differentiation inducers that upregulate neuronal and astrocytic markers in sphere cultures, increase the level of *ATG* mRNAs. G9a binds to the promoters of autophagy (*LC3B, WIPI1*) and differentiation-related (*GFAP, TUBB3*) genes in GSCs. Higher H3K4me3 (an activation mark) and lower H3K9me2 (the repressive mark) levels at the promoters of studied genes were detected in serum-differentiated cells than in sphere cultures. BIX01294 treatment upregulates the expression of autophagy and differentiation-related genes in GSCs. Pharmacological inhibition of autophagy decreases *GFAP* and *TUBB3* expression in BIX01294-treated GSCs suggesting that BIX01294-induced differentiation of GSCs is autophagy-dependent.

Glioblastoma (GBM, WHO grade IV glioma) is the most frequent, primary malignant brain tumor in adults and remains incurable despite aggressive treatments^1^. GBMs are characterized by extensive heterogeneity at the cellular and molecular levels. GBMs contain a rare population of glioma stem-like cells (GSCs, called also glioma-initiating cells) with capacities of self-renewal, multi-lineage differentiation, and resistance to conventional chemotherapy and radiotherapy. GSCs maintain tumor growth, drive tumor progression and cause tumor relapse due to their increased resistance to therapies^2^,^3^,^4^,^5^. GSCs in GBMs share certain characteristics with neural stem/progenitor cells (NSPC) and embryonic stem cells (ESC). Many transcription factors and structural proteins essential for NSPC and ESC function are expressed in GSCs, including NANOG, OCT4 (encoded by the *POU5F1* gene), SOX2, OLIG2, NESTIN and CD133 (Prominin-1)^6^. SOX2, OCT4 and NANOG participate in maintaining self-renewal, proliferation, survival, and multi-lineage differentiation potential of embryonic and somatic stem cells but also GSCs^7^. Epigenome-wide mapping of chromatin states in GBMs identified four core transcription factors, such as POU3F2 (also called OCT7, BRN2), SOX2, SALL2, and OLIG2, which are able to reprogram differentiated tumor cells into GSCs^8^. The differentiated cells loose long-term self-renewal potential *in vitro* and fail to propagate tumors *in vivo*^8^, suggesting that induction of GSC differentiation may be a good strategy to eliminate GSCs in GBM^9^.

Glioma cell differentiation can be induced by anticancer drugs/agents, such as vitamin A and its analogues (including retinoic acid, RA). RA-induced differentiation of GSCs caused anti-migratory, anti-angiogenic and therapy-sensitizing effects *in vitro*, and impaired their tumor-initiating capacity *in vivo*^10^. Unfortunately, most glioblastoma cells are resistant to RA treatment^11^ due to defects of RA signaling pathway components and reduced responsiveness of GSCs^12^. The members of BMP (bone morphogenetic protein) family have been shown to inhibit GSC proliferation and induce GSC differentiation into astroglial and neuronal-like cells, depleting GSC population^13^,^14^,^15^. Although, BMPs promote glial differentiation of GSCs, the epigenetic characteristics of an individual tumor^15^ or secretion of BMP antagonist, Gremlin1, may determine GSC responses to these differentiation-inducing agents^16^.

Activation of the autophagic process may promote GSC differentiation and increase radio- and chemosensitivity^17^,^18^,^19^,^20^. Autophagy is a dynamic recycling process associated with the formation of autophagosomes, the cytosolic double-membrane vesicles that engulf cellular components^21^. Autophagosome formation is controlled by the sequential activation of protein complexes. The ULK1 complex is responsible for autophagy induction, the class III phosphatidylinositol (PtdIns) 3-kinase-BECN1 complex controls the autophagosome nucleation, finally the ATG12–ATG5 and the LC3-I/LC3-phosphatidylethanolamine (PE, LC3-II) complexes participate in extension and closure of the autophagosome membranes^22^. While it is commonly accepted that autophagy plays a key role in cancer, both tumor suppressive and oncogenic activities have been described^23^,^24^. The role of autophagy in cancer stem cells remains unclear. Accumulating evidence suggests that self-renewal, pluripotency and differentiation of stem cells depend on autophagy activity^25^.

Autophagy is tightly regulated by the autophagy-related (*ATG*) genes and epigenetic machinery that involves post-translational modifications of histones^26^. Recently, the histone methyltransferase G9a (also termed EHMT2) which introduces repressive marks: dimethylation of histone H3 lysine 9 (H3K9me2) as well as trimethylation of histone H3 lysine 27 (H3K27me3)^27^ was linked to autophagy^28^. Binding of G9a was found within the promoters of core autophagy genes and pharmacological inhibition or genetic depletion of G9a induced LC3B expression and lipidation in cervical and pancreatic cells^28^. Upon autophagy induction G9a dissociated from the promoters of these genes, which reduced the H3K9me2 repressive histone modifications and increased the H3K9ac active histone marks^28^.

BIX01294, a specific inhibitor of G9a^29^, induced autophagy or autophagy-associated cell death in several tumor cell lines^30^,^31^,^32^,^33^,^34^, but underlying mechanisms have not been explored in glioma cells. Treatment with BIX01294 reduced methylation of H3K9 and H3K27, and at higher concentrations impaired viability of rat C6 glioma cells^35^. BIX01294 is also a potent modulator of stem cell differentiation. BIX01294 pre-treatment improved differentiation of human adipose-derived mesenchymal stem cells into endothelial cells, however it upregulated *POU5F1* and *NANOG* expression^36^. Inhibition of G9a activity with BIX01294 or siRNA significantly increased myogenic differentiation^37^. Bone marrow mesenchymal stem cells differentiated to cardiac-competent progenitors after BIX01294 treatment^38^,^39^. Combination of small molecule inhibitors, BIX01294 and BayK8644 interfered with reprogramming of Oct4/Klf4-transduced mouse embryonic fibroblast into pluripotent stem cells^40^. In GSC-enriched cultures BIX01294 stimulated sphere formation and increased SOX2 and CD133 expression, while overexpression of G9a reversed this effect^41^.

In the present study we sought to examine whether BIX01294 induces autophagy in human glioma cells and how this affects GSC differentiation. We demonstrate that BIX01294 at non-toxic concentrations reduced H3K9me2 and H3K27me3 repressive marks at the promoters of *ATG* genes, inducing autophagy in glioma cells and GSC spheres. The expression of autophagy genes was lower in GSCs than in adherent counterparts. Induction of autophagy in GSCs was associated with the appearance of astrocytic (GFAP) and neuronal (β-tubulin III) differentiation markers. Pharmacological inhibition of autophagy partially abrogated differentiation in BIX01294-treated sphere cultures suggesting that BIX01294 induced differentiation involves autophagy.

## Results

### BIX01294 induces autophagy in glioblastoma cells

**Figure 1:**
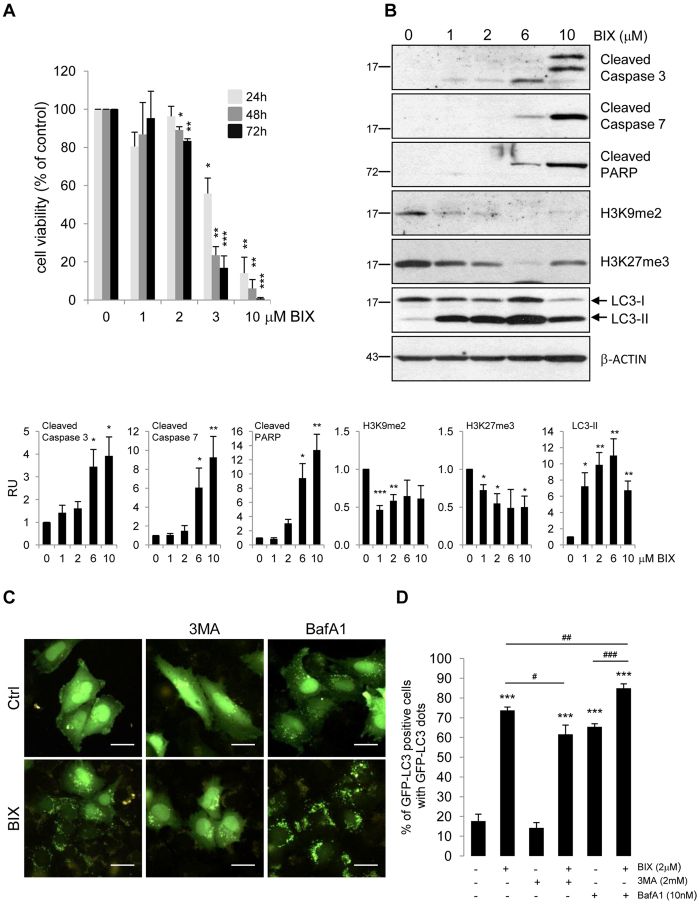
BIX01294 induces autophagy in glioma cells. (**A**) Cell viability of BIX01294 (range = 1–10 μM) treated human LN18 glioma cells was evaluated with MTT metabolism assay. Cells were treated for 24, 48 and 72 h. Results are presented as means ± SEM of three independent experiments. *P < 0.05, **P < 0.01, ***P < 0.001 compared to untreated control cells (Student’s t-test). (**B**) LN18 glioma cells were treated with various concentrations of BIX01294 for 24 h. Western blot analysis was performed using the specified antibodies. Note the increase of apoptosis hallmarks in 6 and 10 μM BIX01294-treated LN18, in contrast to cells exposed to 1 and 2 μM BIX01294, as well as dose-dependent decrease of the level of H3K9me2, H3K27me3 and accumulation of LC3-II in LN18 cells. Equal protein loading was ensured by β-Actin immunodetection. Densitometric analysis of the blots and quantification of the results from three independent experiments is shown; bars represent means ± SEM of the cleaved caspases, cleaved PARP, H3K9me2, H3K27me3 and LC3-II levels normalized to β-Actin and then to the control (untreated cells). The original and full-length blots are presented in Supplementary Fig. S6A. (**C**) Representative microphotographs of cells transfected with GFP-LC3 and treated with BIX01294 (BIX, 2 μM) for 24 hours alone or co-incubated with 3-methyladenine (3MA, 2 mM, 24 h) or 10 nM bafilomycin A1 (BafA1, 4 h). Scale bars represent 20 μm. (**D**) Increase of GFP-LC3-positive cells with GFP-LC3 dots in cells exposed to BIX01294 was reduced following 3MA. Adding of BafA1 increased LC3 punctation. Cells were scored for the presence of GFP-LC3 puncta (more than five dots per cell) among GFP-LC3-transfected cells. A minimum of 100 cells per sample were counted (mean ± SEM, three independent experiments). ***P < 0.001 compared to untreated control cells. ^#^P < 0.05, ^##^P < 0.01 BIX01294-treated cells versus cells co-incubated with 3MA or co-incubated with BafA1, respectively. ^###^P < 0.001 BafA1-treated cells versus cells treated with BIX01294 and BafA1 (compared using t-test).

We examined whether BIX01294 induces autophagy in human glioma cells without affecting cell viability. LN18 glioma cells were exposed to increasing concentrations of BIX01294 (at range = 1–10 μM) for 24, 48 and 72 h and cell viability, apoptotic and autophagic biochemical hallmarks were determined. Cell viability was not significantly affected after exposure to 2 μM BIX01294 for 24 h and only slightly reduced after 48 and 72 hrs. BIX01294 at concentrations 3 and 10 μM reduced cell viability after 24 h by 44% and 86%, respectively (Fig. 1A). Consistently, treatment with higher doses of BIX01294 (6 and 10 μM) for 24 h resulted in accumulation of the cleaved caspase 3, caspase 7 and PARP that evidenced induction of apoptosis (Fig. 1B). Dose-dependent reduction of K9 and K27 methylation of histone 3 was observed in cells exposed to 1, 2 and 6 μM BIX01294. Since 2 μM BIX01294 was sufficient to decrease H3K9me2 and H3K27me3 levels without reducing cell viability (Fig. 1A,B), this concentration was used for further analysis. The most prominent reduction of H3K9me2 and H3K27me3 levels in LN18 cells was observed 24 h after adding 2 μM BIX01294 (Supplementary Fig. S1A).

Dose and time course studies revealed the gradual accumulation of LC3-II, a cellular marker of autophagy upon BIX01294 treatment (Fig. 1B, Supplementary Fig. S1A). BIX01294 treatment caused accumulation of acidic vesicular organelles (AVOs), associated with autophagy in LN18 glioma cells, which was abolished by co-incubation with autophagy inhibitors 3MA (3-methyladenine) or bafilomycin A1 (BafA1) (Supplementary Fig. S1B). The GFP-LC3 plasmid was used to detect autophagic vacuoles in transfected cells. Distribution of GFP-LC3 in untreated cells was diffused and only 20% of the cells contained GFP-LC3 dots (Fig. 1C,D). BIX01294 significantly increased GFP-LC3 punctation up to more than 70% of cells with GFP-LC3 dots. The changes induced by BIX01294 in LN18 glioma cells were partially blocked by 3MA (Fig. 1D, Supplementary Fig. S1C). Co-incubation with BafA1, which prevents fusion of autophagosomes with lysosomes, enhanced BIX01294-induced accumulation of GFP-LC3 punctation (Fig. 1D) and conversion of LC3-I to LC3-II (Supplementary Fig. S1C), which suggests dynamic autophagy in glioma cells upon BIX01294-treatment. Similar phenomenon was observed in the patient-derived L0125 glioma stem-like cells. We found a dose-dependent effect of BIX01294 on cell viability of L0125 cells. Apoptotic hallmarks were observed only in cells exposed to higher doses of the drug (6 and 10 μM). BIX01294 significantly and in a dose-dependent manner reduced the level of H3K9me2, and concomitantly increased the LC3-II level. Moreover, 2 μM BIX01294 enhanced formation of GFP-LC3 puncta which was suppressed by an autophagy inhibitor (3MA) (Supplementary Fig. S2), suggesting that BIX01294 induces autophagy in established cell lines as well as in the patient-derived glioblastoma stem-like cell cultures.

**Figure 2:**
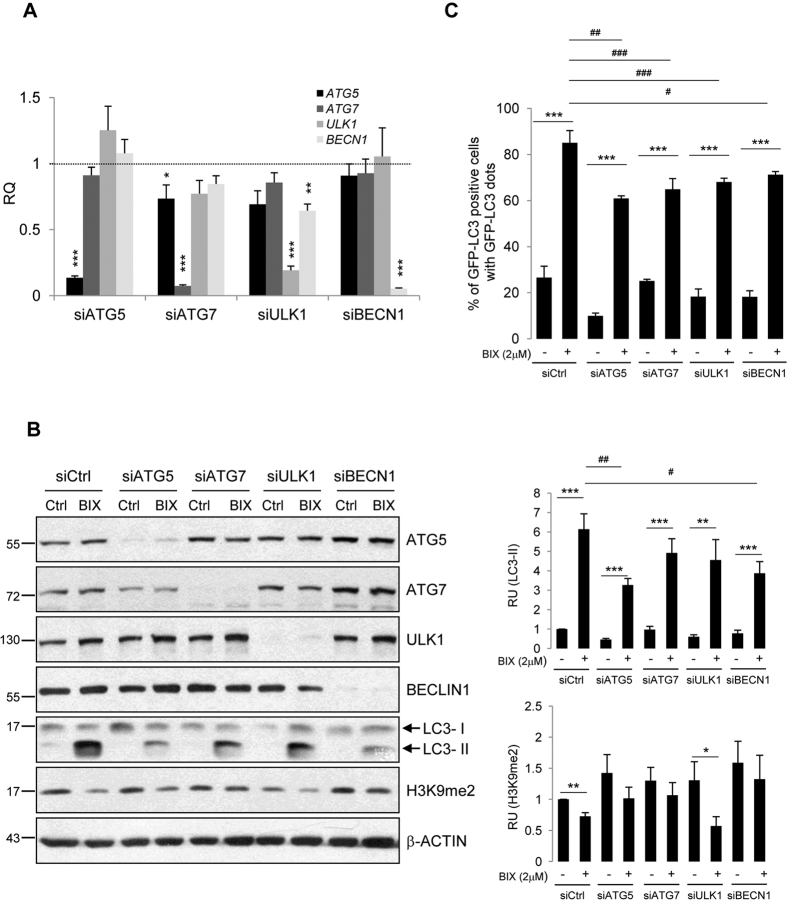
BIX01294-induced autophagy requires *ATG* proteins. Knockdown of *ATG5, ATG7, ULK1* and *BECN1* at the mRNA (**A**) and protein level (**B**) using specific siRNAs. Glioma cells were transfected with *ATG*-targeting siRNA for 48 h, followed by 2 μM BIX01294 for 24 h. (**A**) Each bar represents the mean ± SEM of three independent experiments. Statistical significance calculated to siCtrl-transfected cells (*P < 0.05, **P < 0.01, ***P < 0.001, t-test). (**B**) Immunoblot shows the levels of ATG5, ATG7, ULK1, BECLIN1, H3K9me2 and LC3 in glioma cells. Note the reduction of LC3-II in BIX01294-treated LN18 glioma cells after knockdown of *ATG* genes. The results of densitometric analysis of the blots obtained in three independent experiments are presented (means ± SEM). The levels of H3K9me2 and LC3-II were normalized to β-Actin levels (*P < 0.05, **P < 0.01, ***P < 0.001, t-test). The original and full-length blots are presented in Supplementary Fig. S6B. (**C**) Knockdown of *ATG5, ATG7, ULK1* and *BECN1* affects the accumulation of GFP-LC3 puncta in glioma cells. LN18 cells were transfected with *ATG*-targeting siRNA for 24 h, followed by transfection with GFP-LC3 for 24 h, and followed by 2 μM BIX01294 for the next 24 h. The percentage of GFP-LC3-positive cells was determined as previously described in Fig. 1. Each bar represents the mean ± SEM of three independent experiments. ***P < 0.001 compared to untreated control cells. ^#^P < 0.05, ^##^P < 0.01, ^###^P < 0.001 siCtrl-transfected, BIX01294-treated cells versus cells transfected with *ATG*-targeting siRNA and treated with BIX01294.

The expression of four *ATG* genes was knocked down with specific siRNAs in LN18 glioma cells prior to exposure to 2 μM BIX01294. We found effective (80–95%) silencing of *ATG5, ATG7, ULK1* and *BECN1* at the mRNA and protein levels (Fig. 2A,B). The increase of the lipidated LC3-II level induced by BIX01294 was partially abrogated after knockdown of *ATG* genes. Interestingly, the strongest inhibitory effect was observed in *ATG5* and *BECN1* siRNA-transfected cells, where the BIX01294-induced accumulation of autophagic LC3-II isoform decreased by 1.5-2-fold as compared to the siCtrl transfected cells (Fig. 2B). The levels of H3K9me2 were reduced by BIX01294, and silencing of *ATG5, ATG7* and *BECN1* reversed the BIX01294-induced decrease in H3K9me2 levels. Moreover, the accumulation of cells with GFP-LC3 dots induced by BIX01294 was partially blocked after knockdown of autophagy regulators (Fig. 2C).

### Reduced autophagy in glioma stem-like cells

**Figure 3:**
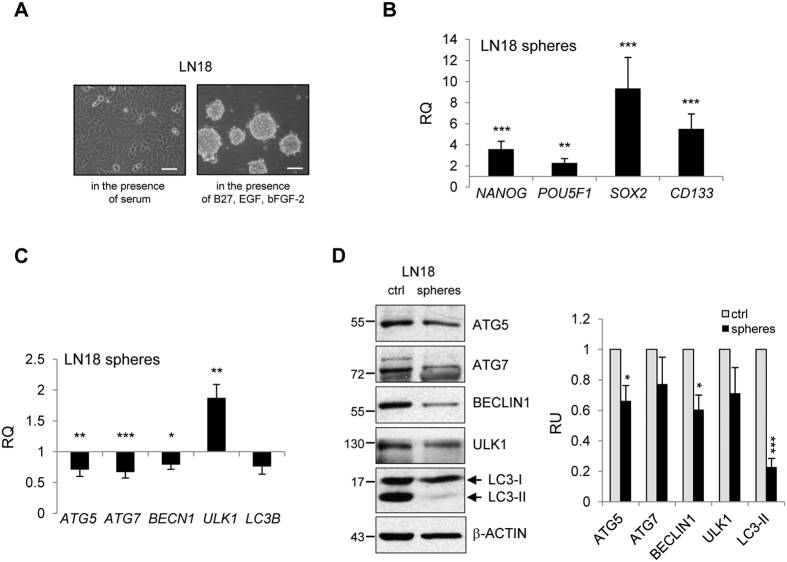
Reduced autophagy in glioma stem-like cells. (**A**) Photographs show morphological changes of LN18 growing in the presence of serum or in the serum-free medium containing cytokines (rh EGF and rh bFGF). Note the formation of neurospheres when the cells are growing in the presence of rh EGF and rh bFGF. Scale bars represent 100 μm. (**B**) Analysis of *NANOG, POU5F1, SOX2* and *CD133* gene expression by qRT-PCR in GSC spheres as compared to the parental cells (n = 3, *P < 0.05, **P < 0.01, ***P < 0.001, t-test). (**C**) *ATG5, ATG7, BECN1, ULK1* and *LC3B* mRNA expression in LN18 spheres was evaluated by qRT-PCR and is shown as a relative fold change in comparison to parental cells (n = 3, *P < 0.05, **P < 0.01, ***P < 0.001, t-test). (**D**) Western blot analysis of autophagy-related proteins in adherent cells (Ctrl) and spheres grown for 7 days. Densitometric analysis was performed; bars show means ± SEM of autophagy-related proteins levels normalized to β-Actin and control (parental LN18 cells) (n = 3, *P < 0.05, ***P < 0.001, t-test). The original and full-length blots are presented in Supplementary Fig. S6C.

To obtain a subpopulation enriched in GSCs, we cultured cells at low density, without serum and with the addition of epidermal growth factor (EGF) and fibroblast growth factor (bFGF). Tumor spheres derived from LN18 cell cultures and glioblastoma-derived WG4 primary cultures were visualized with light microscopy (Fig. 3A, Supplementary Fig. S3A). GCSs-enriched spheres expressed higher levels of pluripotency markers: *NANOG, POU5F1, SOX2* and *CD133* as compared to the adherent tumor cells (Fig. 3B, Supplementary Fig. S3B). Interestingly, tumor spheres expressed the significantly lower level of *ATG5, ATG7, BECN1* and *LC3B* mRNAs (Fig. 3C, Supplementary Fig. S3C). Western blotting revealed the 5-fold lower level of LC3-II and other autophagy-related proteins in cells forming spheres than in the parental cultures (Fig. 3D). These data suggest reduced autophagy in GCSs-enriched spheres.

**Figure 4:**
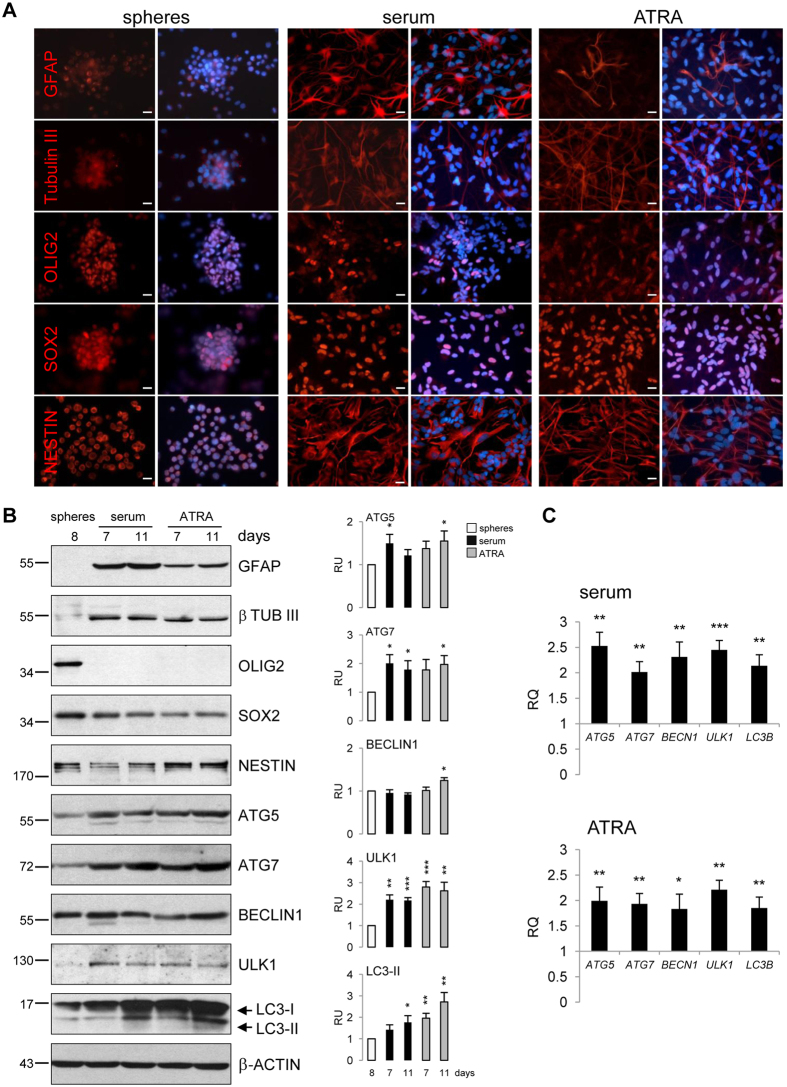
Differentiation of GSCs leads to autophagy induction. (**A**) Immunofluorescence staining of lineage markers GFAP, β Tubulin III and OLIG2, as well as SOX2 and NESTIN before and after differentiation with serum (2% FBS) and ATRA (10 μM ATRA and 2% FBS) of L0125 GSCs line for 11 days. Nuclei were counterstained with DAPI. Scale bars represent 20 μm. (**B**) Western blot analysis was performed using the specified antibodies. Immunoblots show the expression of lineage-specific markers of astrocytes (GFAP) and neurons (β Tubulin III) and decrease of OLIG2 upon exposure of cells to serum and ATRA for 7 and 11 days. Note the accumulation of LC3-II in differentiated cells. β–Actin was used as a loading control. The results of densitometric analysis of the blots obtained in three independent experiments are presented (means ± SEM). The autophagy-related proteins levels normalized to β-Actin and then to the control (spheres) (*P < 0.05, **P < 0.01,***P < 0.001, t-test). The original and full-length blots are presented in Supplementary Fig. S6D. (**C**) Quantitative PCR analysis of autophagy-related genes. Each bar represents x-fold upregulation after differentiation compared with undifferentiated L0125 spheres (n = 3, *P < 0.05, **P < 0.01, ***P < 0.001, t-test).

Serum and all-trans retinoic acid (ATRA) are well known inducers of differentiation in normal and cancer stem cells^10^,^42^. GSCs originating from human GBMs (L0125 and L0627 cell lines^2^,^43^) quickly attached to the cell culture plates and branched out in serum- or ATRA-containing medium. We observed the increased expression of astrocytic (GFAP, glial fibrillary acidic protein) and neuronal (β-Tubulin III) markers in GCSs 7 days upon addition of differentiation media (Fig. 4A,B). Untreated L0125 spheres did not express these proteins, while high levels of OLIG2, SOX2 and NESTIN (neural stem/progenitor markers) were detected. Reduction of OLIG2 levels was particularly visible in ATRA-treated cultures. Increases in GFAP and β-Tubulin III levels, and concomitant decreases in OLIG2 and SOX2 expression were confirmed by Western blotting (Fig. 4B). Similar changes in the expression of differentiation markers were observed in L0627 sphere cultures after treatments (not shown). Transcriptomic analysis, using Affymetrix microarrays, confirmed the prominent increase of *GFAP* mRNA and decrease of *PROM1* and *OLIG2* mRNA levels in serum- differentiated L0627 sphere cultures (Supplementary Fig. S4A). These results verify that serum and ATRA induce differentiation of GBM spheres along astrocytic and neuronal lineages independently of the different genetic background of the cells.

Activation of the autophagic process is associated with GSC differentiation^17^,^18^,^19^,^20^,^44^. Using qRT–PCR we found significantly higher levels of *ATG5, ATG7, BECN1, ULK1* and *LC3B* mRNA in L0125 cultures upon serum- and ATRA-induced differentiation (Fig. 4C). Western blot analysis confirmed accumulation of LC3-II and increases in the levels of ATG7 and ULK1 in differentiated cells, compared to untreated GSCs (Fig. 4B). Additionally, the transcriptomic analysis confirmed increases of *LC3B, BECN1, ATG7, ULK1* and *WIPI2* expression in L0627 cells in the response to serum (Supplementary Fig. S4B). This indicates restoring of autophagic machinery and increased autophagy throughout GSC differentiation.

### BIX01294-induced autophagy leads to differentiation of glioma stem-like cells

**Figure 5:**
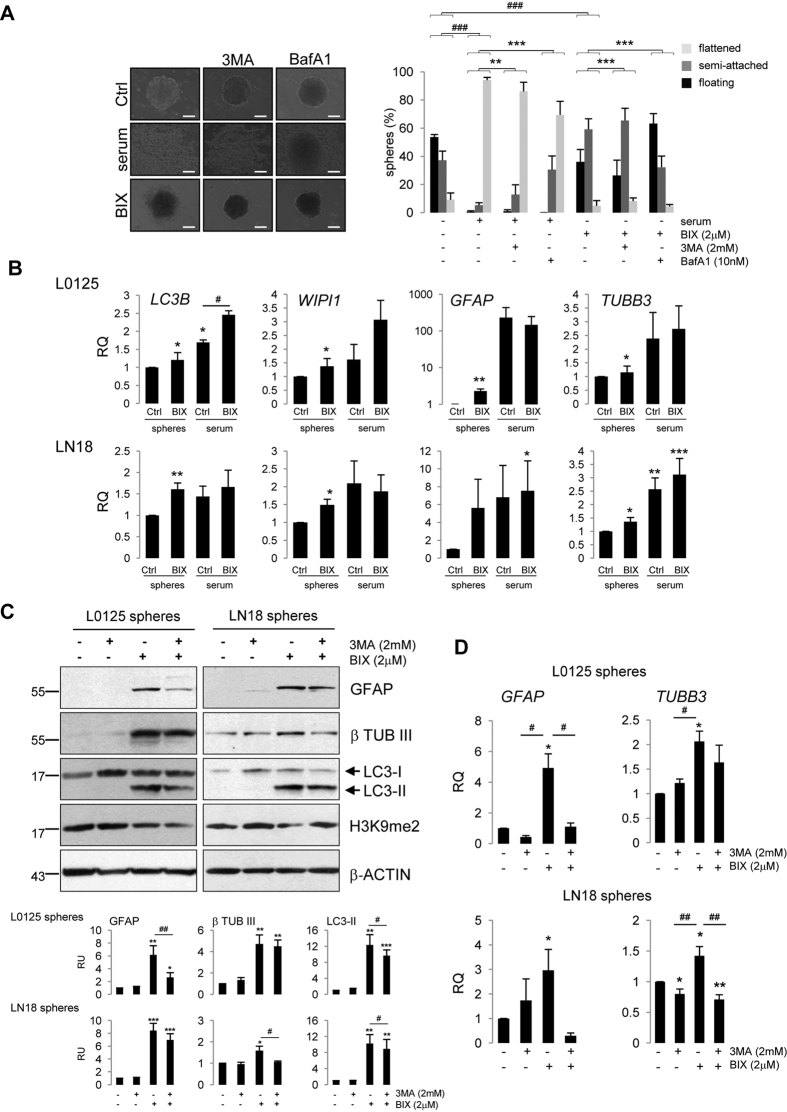
BIX01294-induced autophagy leads to differentiation. (**A**) Representative microphotographs showing morphology changes of L0125 spheres upon serum or 2 μM BIX01294 (BIX) treatment alone or in the presence of 3MA (2 mM) or BafA1 (10 nM). Cells were treated for 48 hours. Scale bars represent 100 μm. Distribution of floating, semi-attached or flattened spheres was determined from four independent experiments. (*P < 0.05, **P < 0.01, ***P < 0.001 compared to appropriate control (untreated, serum- or BIX-treated, Pearson’s Chi-squared test). (**B**) *LC3B, WIPI1, GFAP* and *TUBB*3 mRNA expression in L0125 or LN18 undifferentiated cells (spheres) or differentiated cells (serum), untreated (Ctrl) or treated with 2 μM BIX01294 for 24 h (BIX) was evaluated by qRT-PCR. Each bar represents x-fold upregulation of studied genes with respect to untreated cells forming spheres. (**C**) Immunoblots show the effect of 3MA (2 mM, 24 h) on differentiation markers (GFAP, β Tubulin III) and autophagy marker (LC3) and histone modification (H3K9me2) in control or BIX01294-treated cultures (24 h). Densitometric analysis was performed; bars show means ± SEM of GFAP, β Tubulin III and LC3-II levels normalized to β-Actin (n = 3, *P < 0.05, **P < 0.01, ***P < 0.001, t-test). The original and full-length blots are presented in Supplementary Fig. S6E. (**D**) qRT-PCR analysis of *GFAP* and *TUBB3* gene expression in tumor spheres untreated or treated with 2 μM BIX01294 for 24 h. 3MA (2 mM, 24 h) reduced the gene expression levels upregulated by BIX01294. Each bar represents the mean ± SEM of three independent experiments. *P < 0.05, **P < 0.01 compared to untreated control cells; ^#^P < 0.05, ^##^P < 0.01 compared to BIX01294-treated cells (t-test).

We hypothesized the existence of a causative relationship between BIX01294-induced autophagy and differentiation processes. First, we assessed if L0125 spheres undergo morphological alterations (attachment to the cell culture plates) upon BIX0124 treatment and how serum-induced differentiation process is affected by autophagy inhibitors. Under control conditions 80% of spheres were floating or semi-attached, maintaining their spherical shapes (Fig. 5A). Addition of 3MA or BafA1 for 48 hours had no significant effects (not shown). In contrast, in serum-treated cultures 93% of spheres were flattened and cells branched out. Co-treatment with autophagy inhibitors (3MA, BafA1) significantly decreased the number of flattened spheres, and increased the number of spheres maintaining their spherical shapes (Fig. 5A). L0125 spheres treated with BIX01294 for 48 hours were more attached to the culture plates and began to lose their shapes when compared to untreated spheres (51% semi-attached and 29% floating spheres in BIX01294 treated cells vs. 38% semi-attached and 42% floating spheres in control cells) (Fig. 5A). These effects were slowed down when the cells were co-treated with autophagy inhibitors (BafA1 or 3MA). Especially the increased number of floating spheres and decreased number of semi-attached spheres were observed in cells treated with BIX01294 and BafA1 compared to cells treated with BIX01294 alone (Fig. 5A).

BIX01294 induced the expression of *LC3B* and *WIPI1* along with differentiation markers *GFAP* and *TUBB3* in L0125 and LN18 spheres (Fig. 5B), however less potently than serum. Increased levels of astrocytic (GFAP) and neuronal (β-Tubulin III) markers in BIX01294-treated tumor spheres were confirmed at protein levels (Fig. 5C, Supplementary Fig. S5). It suggests that BIX01294 is a strong inducer of autophagy and differentiation in GSCs.

GFAP and β-Tubulin III expression became less abundant in serum-differentiated spheres upon inhibition of autophagy with 3MA (Supplementary Fig. S5). We tested whether alleviating autophagy could influence BIX01294-induced differentiation process of GSCs. The increased level of the endogenous LC3-II (Fig. 5C) and the number of GFP-LC3 positive cells with puncta were reduced in L0125 GSCs by co-treatment with 3MA (Supplementary Fig. S2C,D). Autophagy inhibitor partially abolished the increase of GFAP expression in L0125 spheres and β-Tubulin III expression in LN18 spheres induced by BIX01294 at both mRNA and protein levels (Fig. 5C,D; Supplementary Fig. S5). In other cases, the effect was not statistically significant, although the direction of changes was maintained. These data indicate that induction of GSC differentiation by BIX01294 depends on transcriptional activation of autophagy genes.

**Figure 6:**
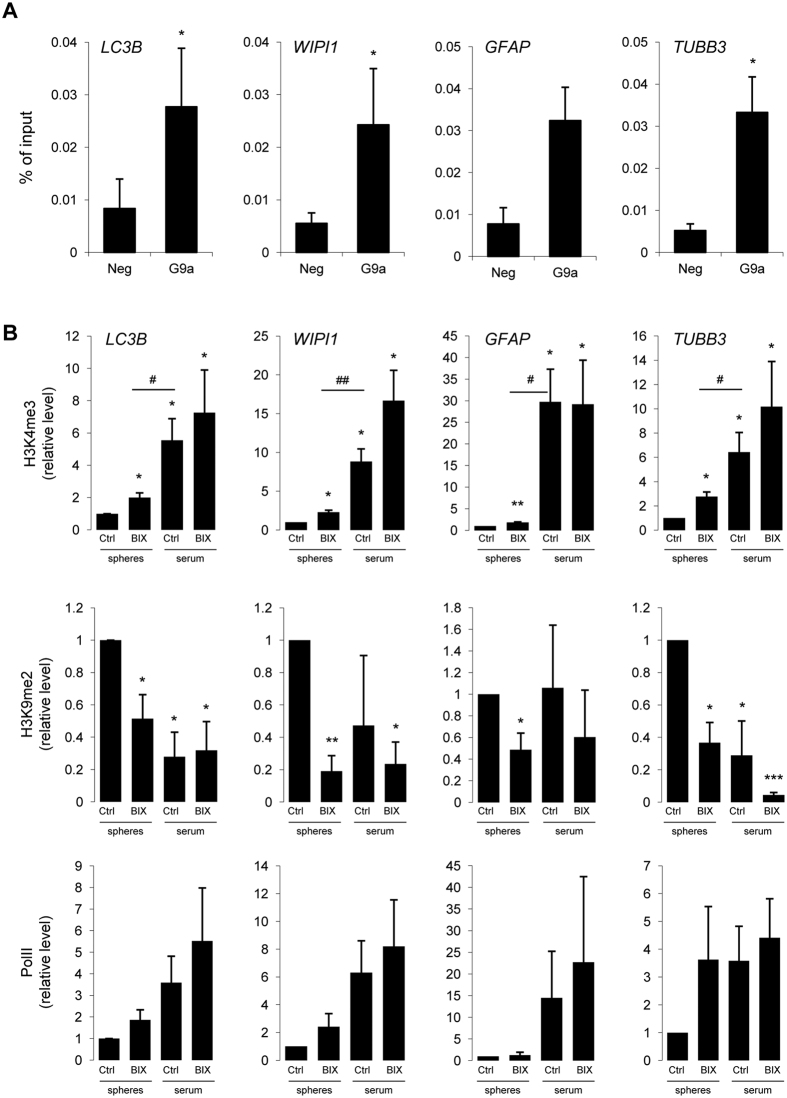
BIX01294 induces chromatin modification of genes crucial for autophagy and differentiation processes. (**A**) Chromatin immunoprecipitation (ChIP)-quantitative polymerase chain reaction (qPCR) analysis of G9a binding to the *LC3B, WIPI1, GFAP* and *TUBB3* promoters in L0125 spheres. Normal rabbit IgG served as a negative control. Results are calculated as % of input, mean ± SEM (n = 4). Statistical significance *p < 0.05 (t-test). (**B**) ChIP-qPCR analysis of histone modifications H3K9me2 (repressive) and H3K4me3 (active) and RNA polymerase II (PolII) binding to the *LC3B, WIPI1, GFAP* and *TUBB3* promoters in L0125 undifferentiated cells (spheres) or adherent cells after differentiation with serum (serum), untreated (Ctrl) or treated with 2 μM BIX01294 for 24 h (BIX). Results are calculated as % of input normalized to the control, mean ± SEM (n = 4). Statistical significance *p < 0.05, **p < 0.01 (t-test).

To get insights into molecular mechanisms of BIX01294-induced differentiation and autophagy processes, we performed a chromatin immunoprecipitation (ChIP)-qPCR analysis for selected histone modifications and binding of G9a to the promoters of genes involved in these two processes. We demonstrated that histone methyltransferase G9a binds to the promoters of autophagy-related (*LC3B, WIPI1*) and differentiation-related genes (*GFAP, TUBB3*) in L0125 spheres (Fig. 6A). Higher levels of H3K4me3 (an active chromatin mark) and lower levels of H3K9me2 (a repressive mark) at the promoters of studied genes were observed in serum-differentiated cells in comparison to spheres (Fig. 6B). The reduction in H3K9me2 at the promoters of *WIPI1* and *GFAP* genes was not statistically significant. BIX01294 induced changes in H3K4me3 and H3K9me2 levels were accompanied by the substantial upregulation of RNA polymerase II binding to the promoters of studied genes (Fig. 6B). This was consistent with the prominent reduction of H3K9me2 in BIX01294-treated LN18 and L0125 spheres (Fig. 5C, Supplementary Fig. S1A, Supplementary Fig. S2B).

**Figure 7:**
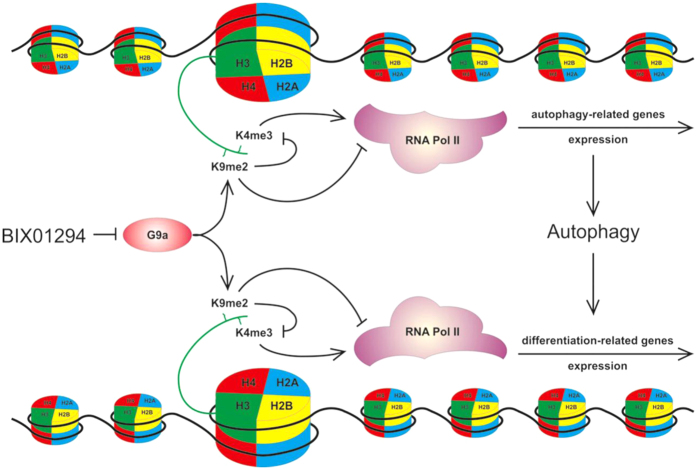
Schematic representation of the proposed molecular mechanism underlying BIX01294 induced autophagy-dependent differentiation of cancer stem cells.

Based on our findings, we propose a model in which BIX01294 inhibits G9a histone methyltransferase in GSCs, which results in reduction of the repressive H3K9me2 marks and the increase in the activation H3K4me3 marks at the promoters of autophagy- and differentiation-related genes. These changes trigger restoration of autophagy components and activation of this process in GSCs, which triggers cell differentiation (Fig. 7).

## Discussion

Accumulating evidence suggests that glioma initiating cells are responsible for glioblastoma propagation, recurrence and resistance to therapy. “Differentiation therapy” is considered as a promising approach to eliminate this cell population in GBM^9^. Epigenetic-based drugs targeting epigenetic enzymes in cancer have been shown to affect GSC differentiation and induce autophagy^19^,^44^. Autophagy is highly active during mammalian development and differentiation, maintains the quality control and cellular homeostasis of terminally differentiated cells^45^, as well as participates in self-renewal, pluripotency and differentiation of stem cells^25^,^46^.

Here, we demonstrated for the first time, that BIX01294 (a G9a inhibitor) reduces H3K9me2 and H3K27me3 levels and induces autophagy in glioma cells. BIX01294, at 5–10 μM concentrations, was reported as an inducer of autophagy–associated cell death in various tumor cells^30^,^31^,^33^,^34^. We confirmed that at high doses 3–10 μM the drug reduced viability of glioma cells and GSC, and induced caspase-dependent cell death which was accompanied by conversion of LC3-I to LC3-II. At a non-toxic dose BIX01294 (2 μM) stimulated LC3-II accumulation and autophagosome formation. BIX01294-induced autophagy was partially blocked by 3-methyladenine and by selective silencing of crucial autophagy genes *ATG5, ATG7, ULK1* and *BECN1*. Similarly, inhibition or knockdown of G9a resulted in increased *LC3B, ATG9B* and *WIPI1* expression, formation of autophagosomes and LC3-II accumulation in cervical cancer HeLa cells and pancreatic cancer SU86.86 cells^28^. We found the increase of *LC3B* and *WIPI1* expression in GSCs in response to BIX01294. However, BIX01294 induced changes could be cell-type specific, as in human colon cancer HCT116 cells the expression of *LC3B, ATG9A, ATG4A*, but not *ATG4B*/*C, ATG7, BECN1, WIPI1* was increased after BIX01294 treatment^34^. The expression of LC3B and ATG3, ATG5, ATG7, ATG12 proteins was also markedly up-regulated after BIX01294 treatment in neuroblastoma cells^31^.

We found induction of autophagy as evidenced by formation of GFP-LC3 puncta, accumulation of LC3-II and the increased expression of *LC3B* in GSCs after BIX01294 treatment. We demonstrate that G9a binds directly to the promoters of *LC3B* and *WIPI1* in GSCs, and BIX01294 enhances H3K4 trimethylation (the active chromatin mark), while reduces the level of the repressive H3K9 dimethylation. This was accompanied by the substantial increase of the RNA polymerase II binding to the promoters of studied genes, including autophagy-related genes. These events corresponded to the higher expression of *LC3B* and *WIPI1* in GSCs. Similar responses to BIX01294 treatment were demonstrated after G9a knockdown in pancreatic cancer cells or cervical cancer HeLa cells^28^.

“Selection by growth requirement” is a method of choice for enrichment in GSCs^47^,^48^. We expanded GSCs from established glioma LN18 cells and cultures derived from GBM specimens (WG4, L0125 and L0627) in serum-free medium with the growth factors (EGF, bFGF). We confirmed higher expression of pluripotency transcription factors (*NANOG, POU5F1, SOX2*) and *CD133* in cells forming tumor spheres. L0125 and L0627 cell cultures, previously characterized as GSCs with different genetic background, displayed self-renewal and tumor-initiating abilities, and multi-lineage differentiation potential^2^,^43^. Herein, we show that in the presence of serum or ATRA these cells upregulated GFAP and β-Tubulin III expression with the concomitant reduction of OLIG2 and SOX2 levels. However, SOX2 was expressed at relatively high level in differentiated L0125 cells, suggesting that its expression in glioma cells may have different, stemness-unrelated functions. We report that these sphere cultures are characterized by lower expression of *ATG* genes and lower level of LC3-II than adherent cultures, which suggests the reduction of autophagy in GSCs. In fact, a couple of previous reports also found the low autophagy activity in GSCs when compared to neural stem/progenitor cells^17^,^49^. CD133 positive GSCs were characterized by higher expression of LC3, ATG5 and ATG12 compared with the CD133 negative cell fraction, whereas no differences were observed in the expression of Beclin1 and ATG7^50^. It is worth noting that glioma cell lines differ in the basal level of autophagy and the higher level of endogenous autophagy was detected in T98G and LN18 the glioblastoma cell lines than in the astrocytoma cell line^51^. Similarly, higher autophagic activity was observed in adult human stem cells and primary breast cancer stem cells as compared to their non-stem counterparts, which correlated with high Beclin1 expression in stem cells^52^,^53^. Paradoxically, autophagy appears to play an important role in adult stem cell maintenance as well as during their differentiation^53^. *Atg7, Becn1, LC3* and *Ambra1*, all involved in autophagy regulation, showed an increased expression during NSC differentiation in neurogenesis of olfactory bulbs in mice^54^. In addition, the increased level of autophagy has been detected in cultured NSC undergoing differentiation *in vitro*^54^. Moreover, induction of autophagy was observed during ATRA-induced neuronal differentiation of neuroblastoma N2a cells^55^ and in serum- and rapamycin-induced GSC differentiation^17^. Chemical or genetic inhibition of autophagy markedly delays or completely blocks the differentiation process of adult, neuronal and glioma stem cells^17^,^53^,^55^.

Interestingly, BIX01294 induced in GSCs similar changes in morphology and biochemical markers as serum, a typical differentiation inducer. We observed the upregulation of GFAP and β-Tubulin III at mRNA and protein levels in LN18 and L0125 sphere cultures upon BIX0194 treatment. Higher H3K4me3 and lower H3K9me2 levels were found at the promoters of *GFAP* and *TUBB3* in spheres treated with BIX01294. Additionally, serum and ATRA administration increased expression of several autophagy-related genes in GSCs. In turn, autophagy inhibitors 3MA and BafA1 prevented or reduced differentiation which confirms the critical role of autophagy in GSCs differentiation. Some data suggest that the autophagy is involved in energy supply during differentiation, restoring ATP levels^54^. To conclude, autophagy appears to play different roles in CSCs. The current hypothetical model describes autophagy as a modulator of differentiation process, but autophagy also plays a crucial role in the origin and maintenance of CSCs besides its many functions in normal embryonic and tissue stem cells^56^.

Finally, question arises regarding the role of autophagy and differentiation process in cancer therapy. Rapamycin-induced autophagy promotes differentiation of GSCs and their radiosensitivity^17^,^18^. A combination of radiotherapy and rapamycin is recommended as a potential therapeutic strategy to enhance current treatments for patients with glioblastoma. In contrast, the induction of autophagy contributes to radioresistance of the CD133+ GSCs and autophagy inhibitors were employed to increase the sensitivity of CD133+ GSCs to γ-radiation^50^. The effect of γ-radiation-induced autophagy on differentiation of the CD133+ GSCs has not been analyzed. BMP2, which promotes differentiation and induces growth inhibition in GBM cells, sensitizes GSCs to temozolomide (the mainstay of anti-glioma chemiotherapy)^57^. SAHA, which was described as promising agent for targeting GSCs through the induction of autophagy and differentiation, reduced tumor growth in xenograft assays^44^. Moreover, drugs that target epigenetic enzymes, including valproic acid (VPA), affected cancer cell differentiation and potentiated efficacy of taxol and nanotaxol in growth arrest of human glioblastoma cells^58^.

Taken together, the results from current investigation and other reports support the idea of GSCs differentiation as a potent strategy to deplete this population in GBM. The role of G9a in regulation of genes related to autophagy and cell differentiation was documented herein. Moreover, we identified autophagy as an upstream process to GSC differentiation, since the inhibition of autophagy, decreased BIX01294-induced cell differentiation. A more detailed understanding of the molecular response of GSCs to BIX01294 may contribute to novel therapeutic approaches to stem cell-targeting cancer therapy.

## Methods

### Reagents and antibodies

Reagent and antibody sources were as follows: acridine orange, ATRA (all-*trans*-retinoic acid), bafilomycin A1 (BafA1), BIX01294 trihydrochloride hydrate, DAPI (4′,6-diamidino-2-phenylindole dihydrochloride), laminin, 3-methyladenine (3MA), MTT (3-(4,5-dimethylthiazol-2-yl)-2,5-diphenyltetrazolium bromide), anti-LC3 (catalog no. L7543) and anti-β-Actin-peroxidase conjugated antibody (catalog no. A3854) (Sigma-Aldrich, Munich, Germany), anti-ATG5 (catalog no. 2630), anti-ATG7 (catalog no. 2631), anti-cleaved caspase 3 (catalog no. 9661), anti-cleaved caspase 7 (catalog no. 9491), anti-cleaved PARP (poly (ADP-ribose) polymerase-1) (catalog no. 9541), anti-SOX2 (catalog no. 3579) (Cell Signaling Technology, Beverly MA, USA), anti-ULK1 (catalog no. sc-33182) (Santa Cruz Biotechnology, Dallas, Texas, USA), anti-Beclin1 (catalog no. 612112), anti-GFAP (catalog no. 556330) (BD Pharmingen San Jose, CA, USA), anti-H3K4me3 (catalog no. 07-473), anti-H3K27me3 (catalog no. ABE44), anti-OLIG2 (catalog no. AB9610), anti-Tubulin beta III isoform (catalog no. MAB1637) (Millipore, Temecula, CA, USA), anti-H3K9me2 (catalog no. ab1220), anti-G9a (catalog no. ab40542) (Abcam, Cambridge, UK), anti-RNA Pol II (catalog no. 39097) (Active Motif, Carlsbad, CA, USA) and anti-NESTIN (catalog no. MAB1259) (R&D Systems, Minneapolis, MN, USA).

### Cell culture and treatments

Human malignant LN18 glioma cells were from American Type Culture Collection and were cultured as previously described^51^. Cells were treated with 1–10 μM BIX01294 for indicated periods of time. Bafilomycin A1 (BafA1, 10 nM) and 3-methyladenine (3MA, 2 mM) were used as inhibitors of autophagy. 10 μM all-*trans-*retinoic acid (ATRA) was used as an inducer of differentiation. All compounds were dissolved in DMSO which was used as a control in corresponding concentrations.

WG4 primary glioma cultures originated from a GBM patient surgical sample. The use of tissue was approved by the Research Ethics Board at Institute of Psychiatry and Neurology in Warsaw, Poland and informed consent was obtained from the patient. All methods were carried out in accordance with the relevant guidelines and regulations. Freshly resected tumour tissue was washed in Hank’s balanced sodium solution (HBSS, Gibco Invitrogen, Basel, Switzerland) and subjected to mechanical and enzymatic dissociation using Neural Tissue Dissociation Kit (Miltenyi Biotec, Bergisch Gladbach, Germany) according to the manufacturer’s instructions. Tumour cells were re-suspended in DMEM/F-12 medium (Gibco Invitrogen, Basel, Switzerland) supplemented with 10% FBS (Gibco Invitrogen, Basel, Switzerland) and plated at a density of 1–2 × 10^6^ cells/60 mm plate. 50% of the fresh medium was replaced every 4 days. Floating red blood cells were removed during medium replacement and subsequent passages of cells. Part of the tumor cells were resuspended in DMEM/F-12 serum-free medium, as described below.

L0125, L0627 GBM GSC lines were obtained and provided by Dr Rossella Galli (San Raffaele Scientific Institute, Milan, Italy)^43^,^59^. These cell lines are characterized by different expression of epidermal growth factor receptor (EGFR) which is a known diagnostic and prognostic marker of human GBM. L0125 cells are EGFR negative, while L0627 cells are EGFR positive. L0125 and L0627 were expanded *in vitro* in selection medium: DMEM/F-12, supplemented with 20 ng/ml rh bFGF and 20 ng/ml rh EGF (see below).

### Sphere cultures and differentiation induction

For sphere forming assay, cells were seeded at a low density (1500 viable cells/cm^2^) onto non-adherent plates and cultured in DMEM/F-12 medium, supplemented with 2% B27 (Gibco Invitrogen, Basel, Switzerland), 20 ng/ml rh bFGF (Miltenyi Biotec, Bergisch Gladbach, Germany), 20 ng/ml rh EGF (StemCell Technologies, Vancouver, BC, Canada), 0.0002% heparin (StemCell Technologies, Vancouver, BC, Canada) and antibiotics (Gibco Invitrogen, Basel, Switzerland). Cells were fed every 3 days by replacing 25% of the medium. After 7 days of culturing the spheres were collected by centrifugation at 110 × g and lysed in Qiagen RLT lysis buffer or lysed in buffer supplemented with complete protease inhibitor cocktail (Roche Applied Science, Indianapolis, IN, USA) for blotting, or fixed with 4% paraformaldehyde (PFA) for immunocytochemistry.

For differentiation experiments, spheres were triturated for a single cell suspension and seeded (2.5 × 10^4^ viable cells/cm^2^) onto laminin-coated plates in the medium without cytokines (rh EGF and rh bFGF) but containing 2% FBS with or without the addition of 10 μM all-*trans-*retinoic acid (ATRA) and were incubated for 7–11 days.

### Cell viability assayed by MTT metabolism

The cell viability was assayed by measuring the conversion of MTT to formazan as previously described^51^,^60^.

### Immunoblotting and immunofluorescence

Western blot analysis was performed as previously described^51^.

Cells grown on coverslips were fixed with 4% paraformaldehyde (PFA). Primary antibodies anti-GFAP (1:1500), anti-β Tubulin III (1:500), anti-OLIG2 (1:500), anti-SOX2 (1:300) were diluted in PBS containing 1% bovine serum albumin and 0.1% Triton X-100 and incubated with cells at 4 °C overnight. After washing, the cells were stained with anti-rabbit or anti-mouse Alexa-555-conjugated secondary antibodies (1:1500) (Life Technologies, Karlsruhe Germany) for 2 h at room temperature. Cells were counterstained with 1 μg/ml DAPI.

### Transfection, RNA interference with siRNA

On-TARGET plus SMART pool siRNA against human *ATG5, ATG7, BECN1* and *ULK1* from Dharmacon (Thermo Scientific, Waltham, MA, USA) were used. Non-targeting siRNA was used as a control (siCtrl).

Glioma cells were cultured in 24-well or 12-well plates at a density of 5 × 10^4^ or 1 × 10^5^ cells per well, respectively and transfected with 30–60 nM siRNA. Transfections were performed using Viromer Blue reagent (Lipocalyx, Halle (Saale), Germany). Gene knockdown efficacy was determined by western blots or RT-PCR. After 48 h the cells were treated with BIX01294 for the next 24 h, then harvested and analyzed by Western blot. Some cells after 24 h from the initial transfection for gene silencing were additionally transfected with a plasmid coding for GFP-LC3 (kindly provided by Prof. Aviva Tolkovsky, University of Cambridge, Cambridge, UK) using Lipofectamine 2000 reagent (Gibco Invitrogen, Basel, Switzerland). 24 h later the cells were treated with BIX 01294 for indicated period of time, then fixed with 3% PFA and GFP-LC3 fluorescence was analyzed.

### Autophagy assay

To detect and quantify the acidic vesicular organelles (AVOs) in cells, the vital staining with acridine orange (1 μg/ml for 15 min) was performed.

The pattern of GFP-LC3 in glioma cells transiently transfected with the fluorescent autophagy marker GFP-LC3 was then analyzed using fluorescence microscopy. Cells were scored for the presence of GFP-LC3 puncta (more than five dots per cell) among GFP-LC3-transfected cells. A minimum of 100 cells per sample were counted by two independent researchers.

LC3 conversion assay. Lipidated LC3-II migrates more rapidly (~16 kDa) than LC3-I (~18 kDa) when proteins are separated by SDS-PAGE. Intensities of LC3-II band were quantified using NIH ImageJ software.

Autophagic flux. To exclude that LC3-II increase is induced by inhibition of lysosomal degradation, treated cells were co-incubated with 10 nM bafilomycin A1 (BafA1) for the last 4 hours.

### qRT-PCR and microarray analysis

Total cellular RNA was extracted using the RNeasy Mini kit (Qiagen, Hilden, Germany) and purified using RNeasy columns according to the manufacturer’s instructions. The integrity of RNA was checked using the Agilent’s 2100 Bioanalyzer. For qRT-PCR total RNA from cells after knockdown of *ATG5, ATG7, BECN1, ULK1* genes was used to synthesize cDNA by extension of oligo(dT)_15_ primers with M-MLV reverse transcriptase (Sigma-Aldrich, Munich, Germany). Real-time PCR amplifications were performed in duplicates on cDNA equivalent to 18.75 ng RNA in 10-μl reaction volume containing 2× SYBR GREEN FAST PCR Master Mix (Applied Biosystems, Darmstadt, Germany) and a set of primers. *NANOG* and *POU5F1*(*OCT3*/*4*) were from Qiagen (Hilden, Germany) QT01844808 and QT00210840, respectively. Sequences of primers for other genes are in the Supplementary Table S1. 18S rRNA was used as an internal standard reference. Data were analyzed by the Relative Quantification (^ΔΔ^Ct) method using StepOne Software (Applied Biosystems, Darmstadt, Germany). The expression of each product was normalized to 18S rRNA and is shown as the ratio of the target gene to 18S gene expression, calculated by 2^−ΔΔCt^.

For Microarray analysis human GBM CSC line (L0627) was used. Cells cultures in stem cell-specific medium or medium supplemented with 2% serum for 7 days were analyzed. The hybridizations were performed using Human Genome U219 Array Plate (Affymetrix, Santa Clara, CA, USA). Microarray data were normalized with RMA method. We combined the data set for each condition (n = 3) and analyzed in pairs coming from the same cell culture passage.

### Chromatin Immunoprecipitation (ChIP)

For ChIP-qPCR experiments, approximately 1 × 10^7^ L0125 cells forming spheres or serum-differentiated cells were treated with 2 μM BIX01294 for 24 h or left untreated. Fixation with 1% formaldehyde, sonication, and immunoprecipitation were performed with components of the ChIP IT kit according to the manufacturer’s instructions (Active Motif, Carlsbad, CA, USA). Each sample was immunoprecipitated with 1 μg of one of the following antibodies: anti-H3K9me2, anti-H3K4me3, anti-G9a or anti-RNA Pol II. Normal rabbit IgG (NI01) from Calbiochem (Darmstadt, Germany) served as the control immunoprecipitation antibody. Real-time PCR amplifications were performed in duplicates with immunoprecipitated DNA as the template in a reaction volume of 10 μl; the reaction contained 2× SYBR GREEN FAST PCR Master Mix (Applied Biosystems, Darmstadt, Germany) and primers to a specific promoter. The sequences of the primers used for ChIP-qPCR are located at or around transcription start sites (TSS) of the indicated genes and are presented in Supplementary Table S1.

### Statistical analysis

Data were analyzed by Student’s t-test or chi-square test and are presented as mean ± SEM. *P < 0.05, **P < 0.01, ***P < 0.001 were considered statistically significant.

## Additional Information

**How to cite this article**: Ciechomska, I. A. *et al*. BIX01294, an inhibitor of histone methyltransferase, induces autophagy-dependent differentiation of glioma stem-like cells. *Sci. Rep.* **6**, 38723; doi: 10.1038/srep38723 (2016).

**Publisher's note:** Springer Nature remains neutral with regard to jurisdictional claims in published maps and institutional affiliations.

## Supplementary Material

Supplementary Information
