# Supplementary material for: BIX01294, an inhibitor of histone methyltransferase, induces autophagy-dependent differentiation of glioma stem-like cells
Source: Sci Rep. 2016 Dec 9;6:38723. doi: 10.1038/srep38723 (PMC5146656; doi:10.1038/srep38723)

BIX01294, an inhibitor of histone methyltransferase, induces autophagy-dependent differentiation of glioma stem-like cells

Iwona Anna Ciechomska*[1], Piotr Przanowski[1], Judyta Jackl[1], Bartosz Wojtas [1], Bozena Kaminska[1]
[1]Laboratory of Molecular Neurobiology, Neurobiology Center, Nencki Institute of Experimental Biology of Polish Academy of Science, 3 Pasteur Str., 02-093 Warsaw, Poland

*Corresponding author: Iwona Anna Ciechomska
Neurobiology Center
Nencki Institute of Experimental Biology of Polish Academy of Science
3 Pasteur Str., 02-093 Warsaw, Poland
Fax: (48 22) 8225342
Phone: (48 22) 5892328
E-mail: i.ciechomska@nencki.gov.pl

Supplementary Table S1

A

Sequences of the primers used in this work

ATG5_qPCR	TGAAATGCAGAAAAAAGATCACAAG	TGCAGGATATTCCATGAGTTTCC
ATG7_qPCR	TGAACCTCAGTGAATGTATGGACCCT	TGACAGACACAACCTTGTCCAAGTCTA
BECN1_qPCR	GGCGAAACCAGGAGAGACCCAGG	GTGGACATCATCCTGGCTGGGGG
ULK1_qPCR	GGTCCCCGCGCAGTTTCCAG	GTGGCTCTCCAAGCCCGCAG
LC3B_qPCR	TCCGACTTATTCGAGAGCAGC	AAGCTGCTTCTCACCCTTGT
WIPI1_qPCR	TCCACGGTGCCAGGTTATTC	CCCTTCTGATTTCCACGGCA
SOX2_qPCR	GGGGAAAGTAGTTTGCTGCC	CGCCGCCGATGATTGTTATT
CD133_qPCR	TGGATGCAGAACTTGACAACGT	ATACCTGCTACGACAGTCGTGGT
GFAP_qPCR	TCCTGGAACAGCAAAACAAG	CAGCCTCAGGTTGGTTTCAT
TUBB3_qPCR	GTACGTGCCTCGAGCCATTCT	CGTGTAGTGACCCTTGGCCC
18S_qPCR	CGGACATCTAAGGGCATCAC	AACGAACGAGACTCTGGCAT
LC3B_ChIP	AGGAGATACAAGGGAAGTGGCT	TTGAAGGTCTTCTCCGACGGCAT
WIPI1_ChIP	TTGCCGGACTGAAACCAGCAGAT	TAACGCGGCTCTCCATTGGACAAA
GFAP_ChIP	GTCCTCTTGCTTCAGCGGTC	CAGCCCAGCTATGGGGAGAG
TUBB3_ChIP	CCCTGCGAACCTGCAACAA	AGAGGTGCAGGCGAGAGTC

B

The localization of the primers used for ChIP-qPCR analysis

LC3B_ChIP	+114bp +257bp from TSS
WIPI1_ChIP	-418bp -271bp from TSS
GFAP_ChIP	-206bp -57bp from TSS
TUBB3_ChIP	+271bp +362bp from TSS

Supplementary Figure S1

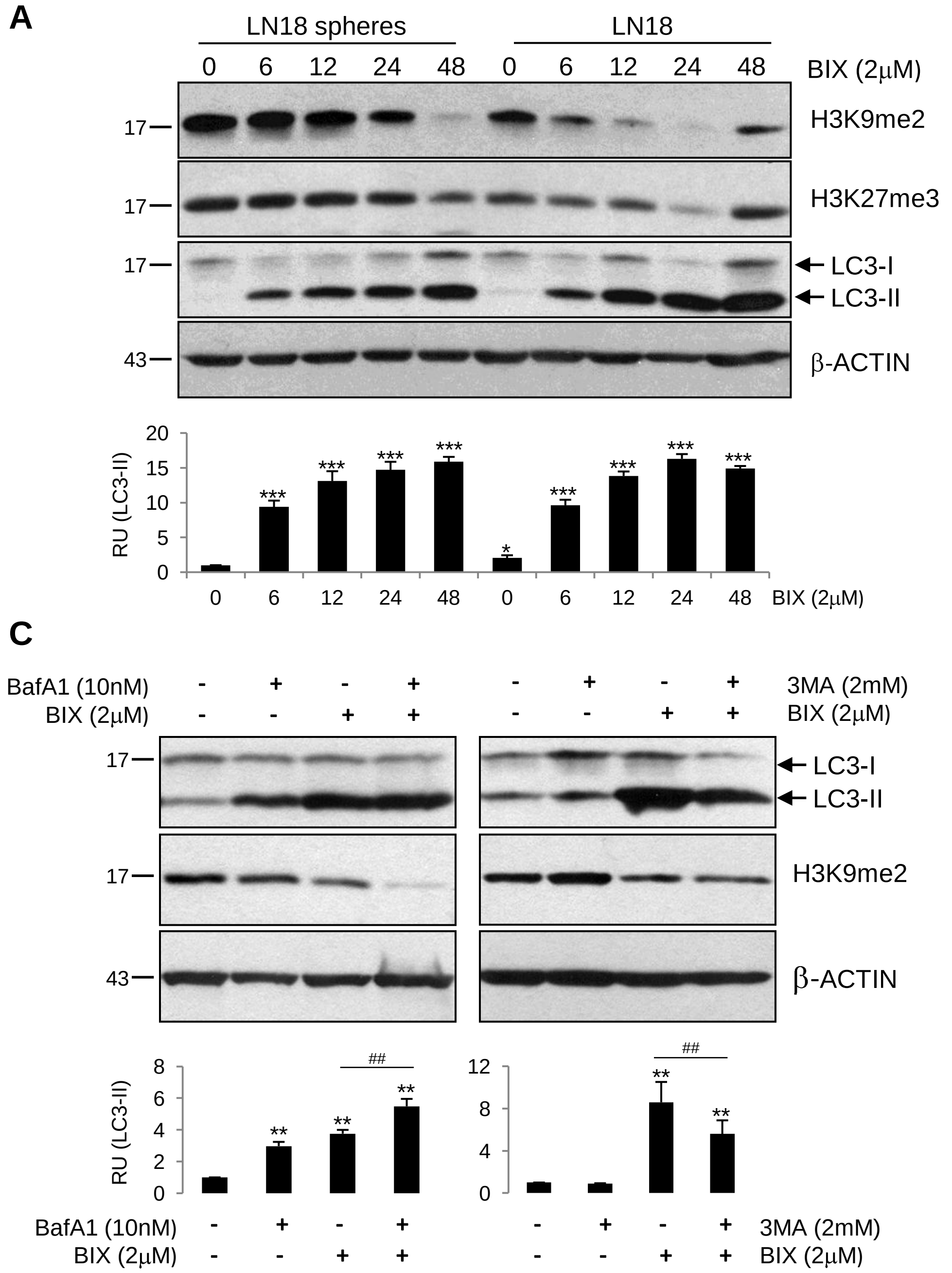

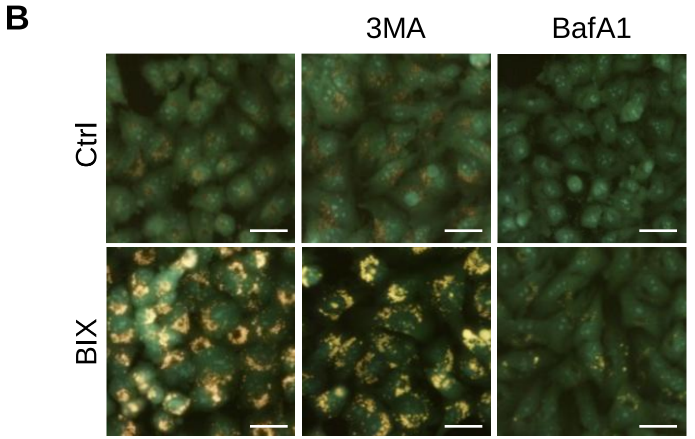

Supplementary Figure S1. BIX01294 induces autophagy in LN18 glioma cells and cells forming spheres. **(A)** Representative immunoblots show H3K9me2, H3K27me3 and LC3 levels in LN18 glioma cells and LN18 spheres after exposure to 2 μM BIX01294 at various time points. Densitometric analysis was performed; bars show means +/- SEM of LC3-II level normalized to β-Actin and control (untreated spheres) (n=3, *P<0.05, ***P<0.001, t-test) **(B)** Fluorescent micrographs show the formation of acidic vesicular organelles (AVOs) in LN18 glioma cells treated with BIX01294 (2 μM) for 24 hours and stained with acridine orange (1 mg/ml for 15 min). Adding of 3-methyladenine (3MA, 2 mM, 24 h) or co-incubation with 10 nM bafilomycin A1 (BafA1) for the last 4 hours significantly reduced AVO formation (decrease of red color intensity). Representative results of two independent experiments are shown. Scale bars represent 200 μm. **(C)** Representative immunoblots show the effects of BafA1 (10 nM, 4 h) (left panel**)** and 3MA (2 mM, 24 h) (right panel) and BIX01294 (2 μM) on LC3 and H2K9me2 in LN18 glioma cells. Densitometric analysis was performed; bars show means +/- SEM of LC3-II level normalized to β-Actin and control (untreated cells) (n=3, **P<0.01, t-test)

Supplementary Figure S2

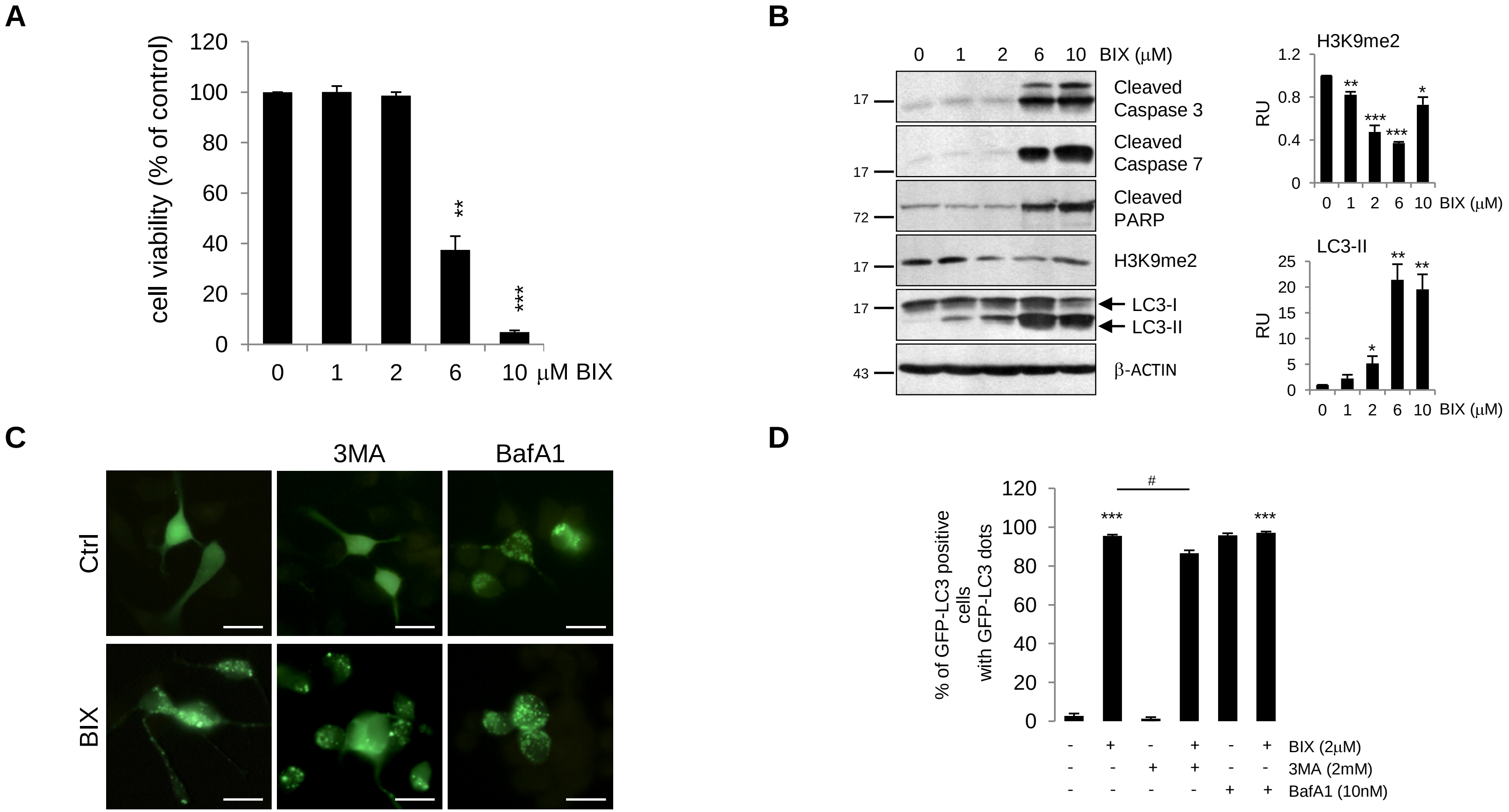

Supplementary Figure S2. BIX01294 induces autophagy in L0125 cell line growing as adherent GSCs **(A)** Cell viability of BIX01294 (range=1-10 μM) treated human L0125 glioma cells was evaluated with MTT metabolism assay. Cells were treated for 24 h. Results are presented as means +/- SEM of three independent experiments. **P<0.01, ***P<0.001 compared to untreated control cells (Student's t-test) **(B)** L0125 cell line growing as adherent GSCs were treated with various concentrations of BIX01294 for 24 h. Western blot analysis was performed using the specified antibodies. Equal protein loading was ensured by β-Actin immunodetection. Densitometric analysis was performed; bars show means +/- SEM of H3K9me2 and LC3-II levels normalized to β-Actin and control (untreated cells) (*P<0.05, **P<0.01 ,***P<0.001, t-test). **(C)** Representative microphotographs of L0125 cells transfected with GFP-LC3 and treated with BIX01294 (BIX, 2 μM) for 24 hours alone or co-incubated with 3-methyladenine (3MA, 2 mM, 24 h) or 10 nM bafilomycin A1 (BafA1, 4 h). Scale bars represent 20 μm. **(D)** Increase of GFP-LC3-positive cells with GFP-LC3 dots in L0125 cells exposed to BIX01294 was reduced following 3MA. Each bar represents the mean +/- SEM of three independent experiments. ***P<0.001 compared to untreated control cells. #P<0.05 BIX01294-treated cells versus cells co-incubated with 3MA (compared using t-test).

Supplementary Figure S3

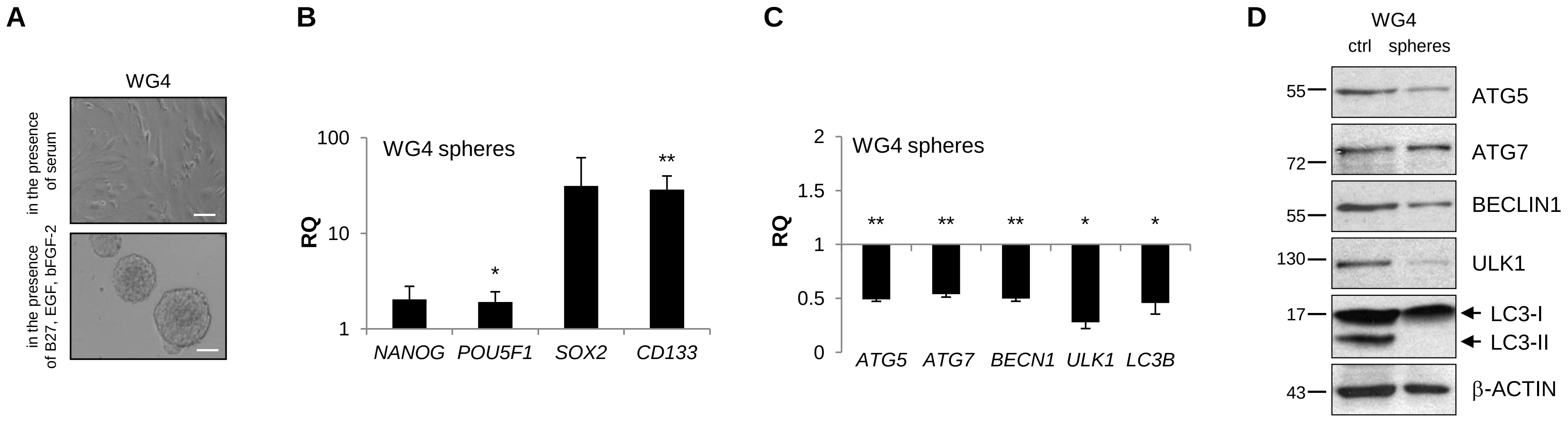

Supplementary Figure S3. Reduced autophagy in primary glioma stem-like cells. **(A)** Photographs show morphological changes of GBM patient derived-WG4 cells growing in the presence of serum or in the serum-free medium containing cytokines (rh EGF and rh bFGF). Note the formation of neurospheres when the cells are growing in the presence of rh EGF and rh bFGF. Scale bars represent 100 μm. **(B)** Analysis of *NANOG*, *POU5F1*, *SOX2* and *CD133* gene expression by qRT-PCR in GBM neurospheres as compared to the bulk tumor cells (n=3, *P<0.05, **P<0.01, t-test). **(C)** *ATG5*, *ATG7*, *BECN1*, *ULK1* and *LC3B* mRNA expression in WG4 spheres was evaluated by qRT-PCR and is shown as a relative fold change in comparison to bulk tumor cells (n=3, *P<0.05, **P<0.01, t-test). **(D)** Western blot analysis of autophagy-related proteins in adherent cells (Ctrl) and spheres grown for 7 days. Similar results were obtained in three independent experiments.

Supplementary Figure S4

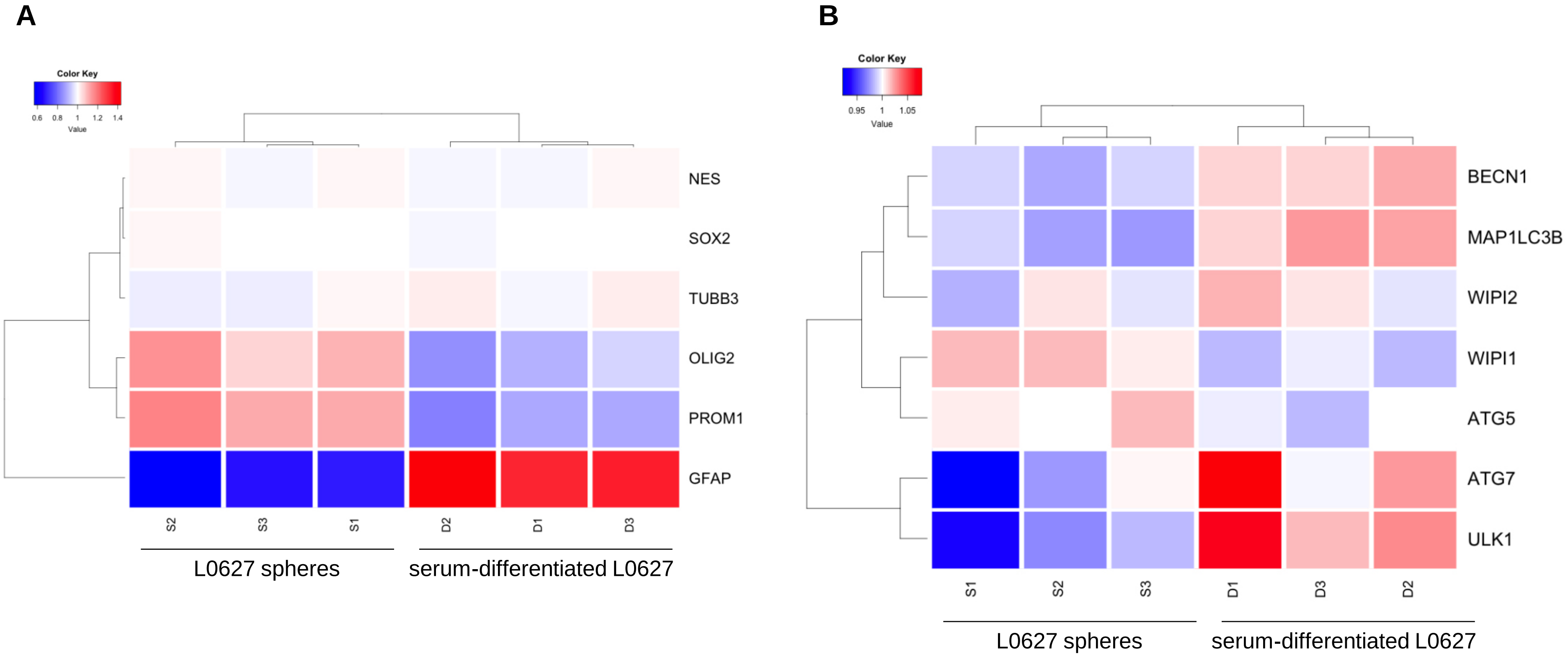

Supplementary Figure S4. Genome wide gene expression profiles of GSCs and serum-differentiated cells. Microarray analysis of gene expression in L0627 spheres and serum-differentiated L0627 cells (S1-3, samples from cells forming spheres) (D1-3, cells differentiated with 2% serum for 7 days). Heat maps shows relative gene expression normalized in pairs (1-3) coming from the same cell culture passage. **(A)** Heat map representation of RMA normalized microarray expression data for "stemness"- and differentiation-related genes in L0627 cells. **(B)** Different autophagy-related genes expression in spheres and differentiated cells shown in the heat map.

Supplementary Figure S5

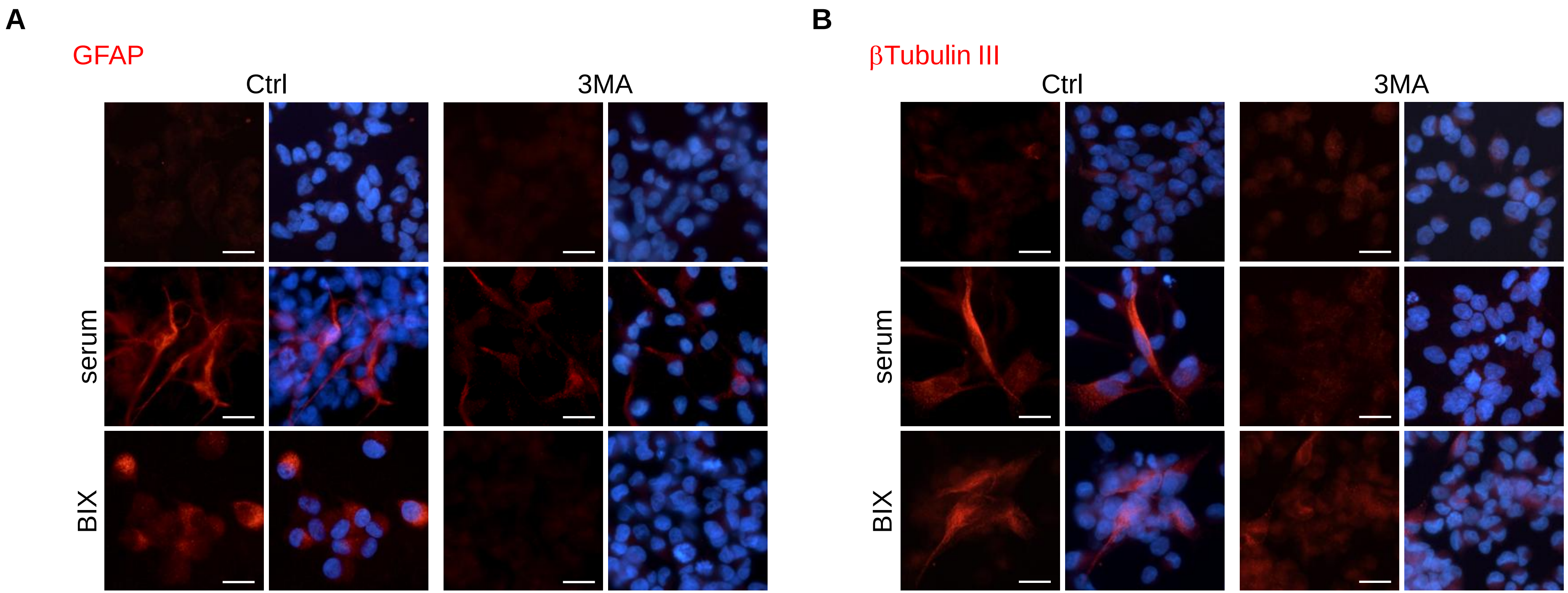

Supplementary Figure S5. Autophagy modulates GSCs differentiation. Increased expression of differentiation markers GFAP **(A)**, β Tubulin III **(B)** upon serum (2% FBS, 48 h) and BIX (2 μM BIX01294, 48 h) treatment in L0125 cell line growing as adherent GSCs (Ctrl). Co-incubation with 2 mM 3-methyladenine (3MA,) or 10 nM bafilomycin A1 (BafA1) significantly reduced the expression of lineage markers (decrease of red color intensity). Representative results of two independent experiments are shown. Scale bars represent 20 μm.

Supplementary Figure S6

Supplementary Figure S6. Full-length blots used in the main figures

A

Full-length blots used in the main Figure 1

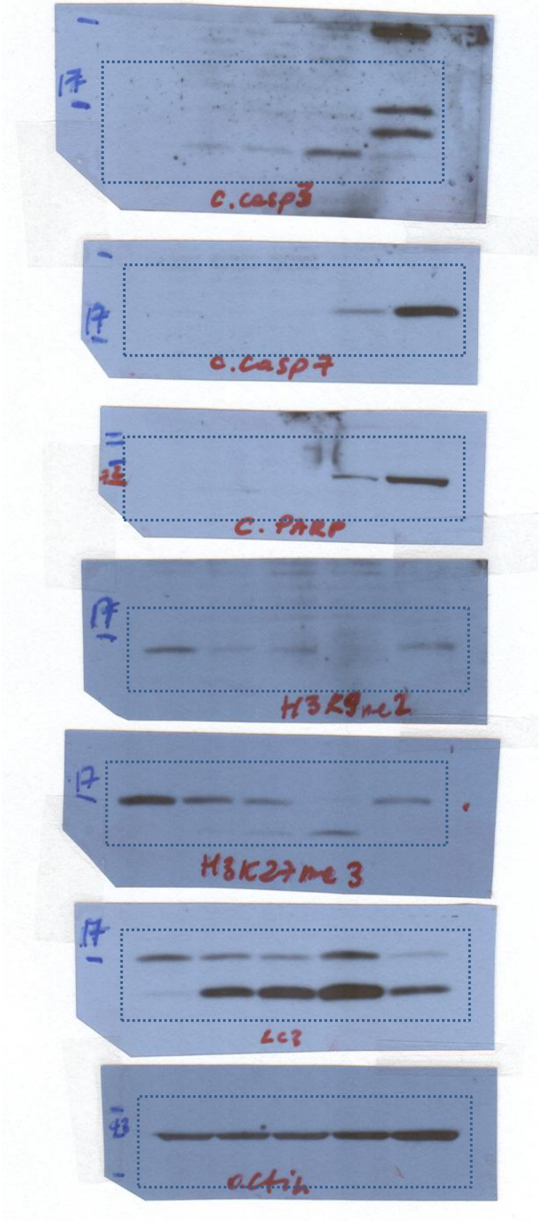

B

Full-length blots used in the main Figure 2

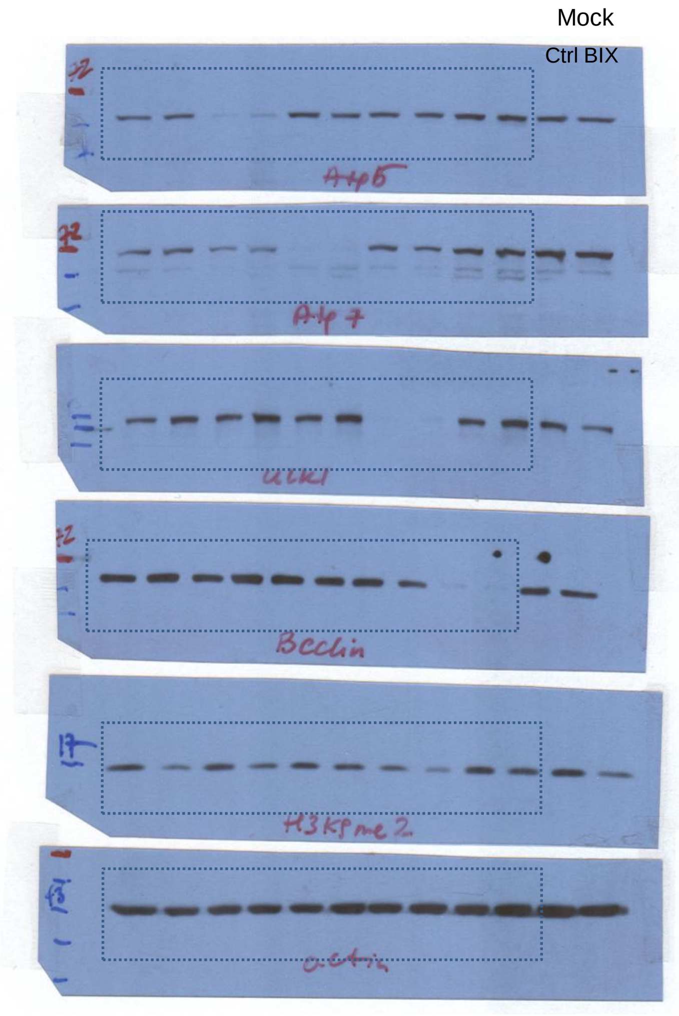

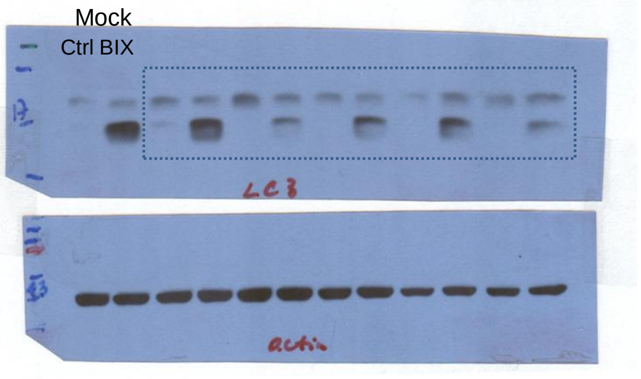

Supplementary Figure S6

C

Full-length blots used in the main Figure 3

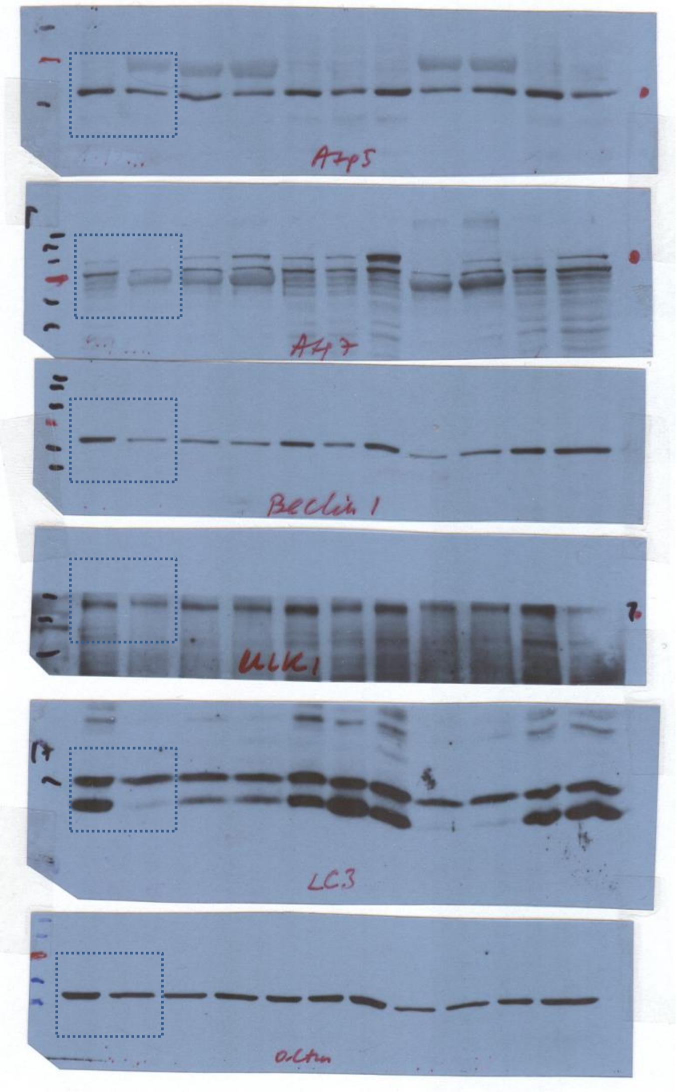

D

Full-length blots used in the main Figure 4

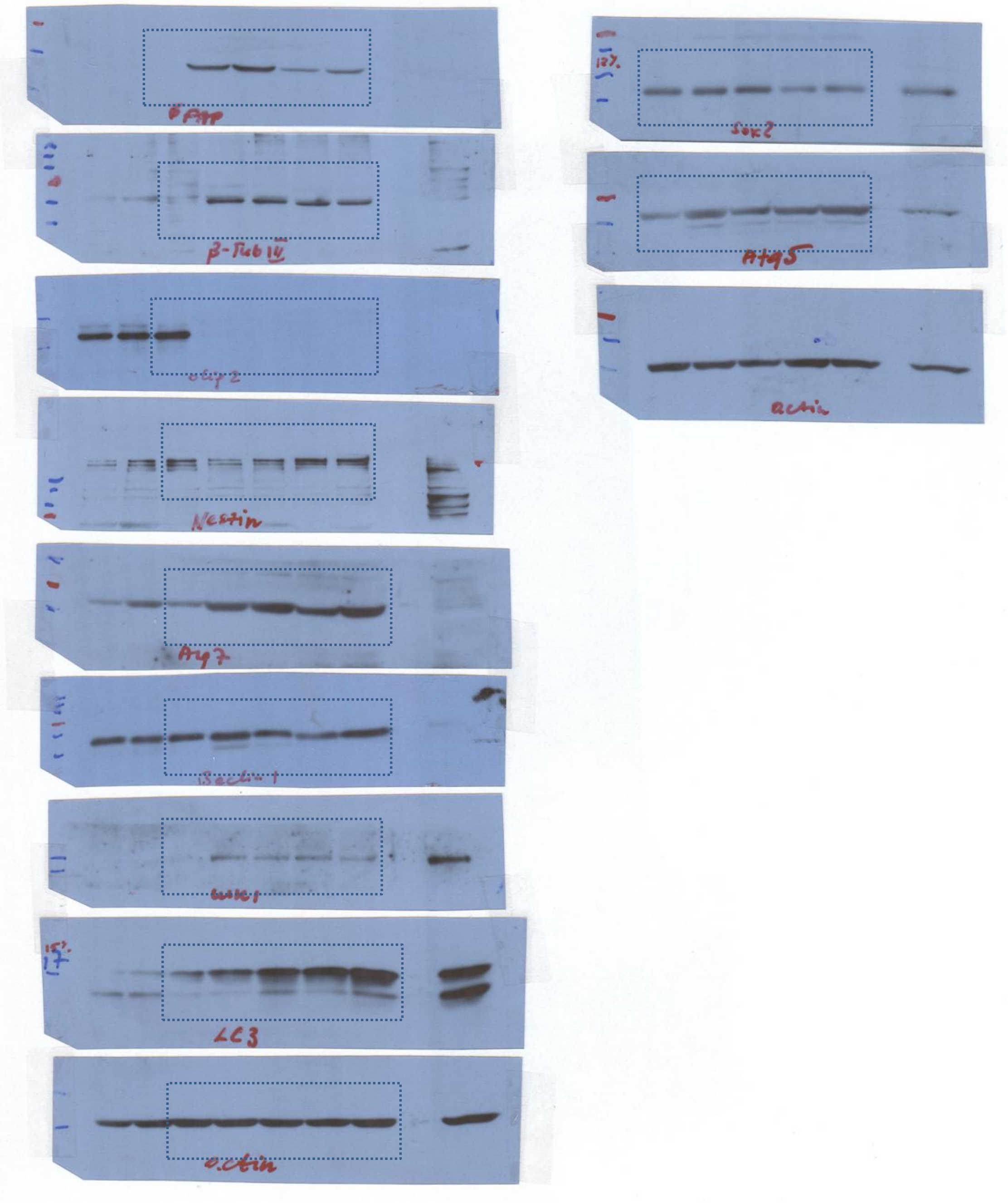

Supplementary Figure S6

E

Full-length blots used in the main Figure 5

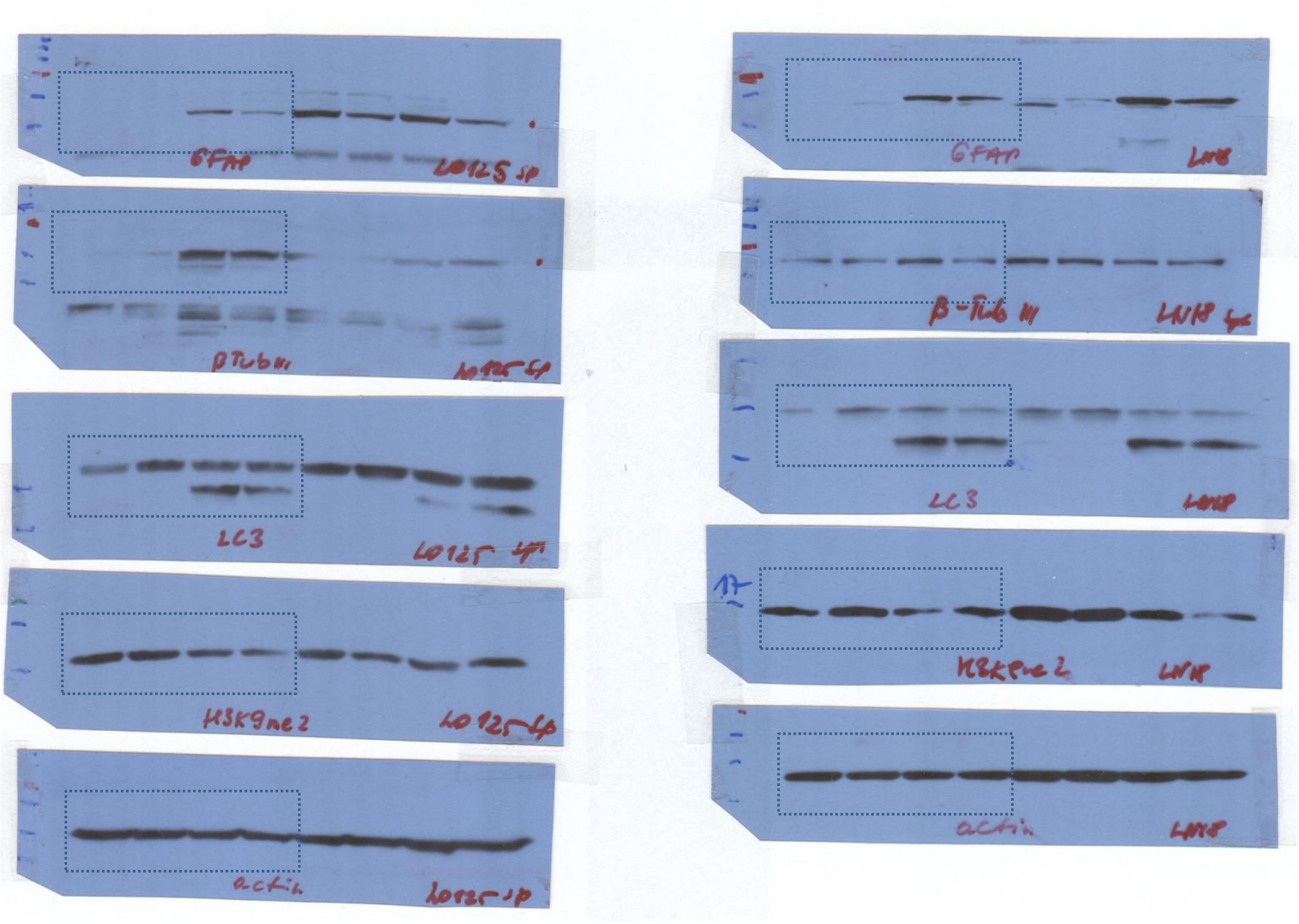

Supplement: Supplementary Information [file srep38723-s1.pdf]
